# Neurovascular uncoupling in type 2 diabetes mellitus: multimodal MRI reveals the association between hippocampal and subcortical structures dysfunction and cognitive decline

**DOI:** 10.3389/fneur.2026.1937804

**Published:** 2026-10-02

**Authors:** Wenjie Chen, Mingrui Li, Weiyin Vivian Liu, Yangye Zheng, Zhongshao Zhang, Wenfeng Huang, Shutong Huang, Hailing Zhou

**Affiliations:** 1 Central People's Hospital of Zhanjiang, Zhanjiang, China; 2 Zhanjiang First Hospital of Traditional Chinese Medicine, Zhanjiang, China; 3 General Electric Company Healthcare, Magnetic Resonance Research, Beijing, China

**Keywords:** type 2 diabetes mellitus, multimodal MRI, neurovascular uncoupling, hippocampal, subcortical structures

## Abstract

**Objective:**

To investigate the efficiency of neurovascular coupling (NVC) in hippocampal and subcortical structures in type 2 diabetes mellitus (T2DM) and its relations with cognition and glycemic control, and to characterize multimodal MRI features of T2DM-related cognitive decline.

**Methods:**

Forty-nine T2DM patients and 43 healthy controls underwent montreal cognitive assessment (MoCA), digit span (DST), clock drawing (CDT), and fasting blood glucose (FBG)/glycated hemoglobin (HbA1c) measurement. High-resolution T1-weighted structural images, three-dimensional arterial spin labeling (3D-ASL) perfusion images, and resting-state functional MRI (rs-fMRI) were acquired on a 3T scanner. Volumes, cerebral blood flow (CBF), fractional amplitude of low-frequency fluctuations (fALFF), regional homogeneity (ReHo), degree centrality (DC), and NVC were calculated for bilateral hippocampus, amygdala, anterior thalamus, caudate, nucleus accumbens, putamen, and globus pallidus. Partial correlations assessed associations between imaging metrics, glycemic parameters, and MoCA scores.

**Results:**

T2DM had significantly lower MoCA (*P* < 0.05) but no differences in DST or CDT. Right anterior thalamic volume was reduced. CBF decreased in bilateral hippocampus, bilateral anterior thalamus, and right amygdala. ReHo reduced in right anterior thalamus and left amygdala. NVC decreased in left hippocampus and left amygdala. No basal ganglia differences were found. MoCA negatively correlated with FBG (*r* = −0.405) and positively with right thalamic volume (*r* = 0.311). HbA1c and FBG negatively correlated with left anterior thalamic CBF (*r* = −0.344 and −0.367, *P* < 0.05).

**Conclusions:**

T2DM shows decreased perfusion, reduced regional synchronization, and neurovascular uncoupling in limbic-subcortical structures including hippocampus, anterior thalamus, amygdala, while basal ganglia motor circuits are preserved. Multimodal MRI metrics may provide imaging targets for early identification and intervention.

## 1 Introduction

Type 2 diabetes mellitus (T2DM) is a metabolic disorder characterized by chronic hyperglycemia, insulin resistance, and low-grade inflammation, with its central nervous system complications garnering increasing attention. Epidemiological evidence indicates that the risk of cognitive decline in patients with T2DM is increased by 50%−100% compared with healthy populations (1); however, the neural mechanisms underlying cognitive decline remain incompletely understood.

Conventional structural magnetic resonance imaging (MRI) studies have primarily identified T2DM-related brain atrophy (2), whereas functional and perfusion imaging have revealed abnormalities in regional brain activity and cerebral blood flow (3). Nevertheless, structural, functional, and perfusion alterations do not occur independently; they may be interconnected through a common pathophysiological bridge—neurovascular coupling (NVC). NVC refers to the physiological mechanism by which local cerebral blood flow (CBF) rapidly matches increased neuronal activity, and its integrity is essential for maintaining cerebral energy metabolism and information processing efficiency (4). However, with the increasing prevalence of resting-state functional magnetic resonance imaging (rs-fMRI), researchers have extended this dynamic coupling concept to the steady-state domain. Currently, resting-state neurovascular coupling is operationally defined as the voxel-wise correlation between resting-state cerebral blood flow (CBF) and the fractional amplitude of low-frequency fluctuations (fALFF). This resting-state metric reflects the steady-state efficiency of metabolic support for spontaneous neural activity and has been applied in clinical neuroimaging studies as a valid proxy for assessing neurovascular integrity (5–7).

In the chronic hyperglycemic milieu of T2DM, oxidative stress, endothelial dysfunction, and neurovascular unit damage can impair NVC (8–10). However, previous studies have predominantly focused on cortical regions, with investigations of the hippocampus and subcortical structures remaining relatively fragmented and lacking integrative analyses of multimodal metrics. Key limbic structures such as the hippocampus and amygdala, along with subcortical nuclei including the thalamus, play central roles in cognition, emotion, and memory. As an information integration hub, the anterior thalamic nuclei participate in consolidation of episodic memory via the Papez circuit (11); the hippocampus is the core structure for memory formation (12); and the amygdala is responsible for emotional regulation and emotional memory encoding (13). These regions have high energy demands, receive blood supply from perforating arterioles, and are particularly vulnerable to microvascular pathology (14, 15). Previous studies have reported volume reduction or hypoperfusion in the hippocampus, thalamus, and other regions in T2DM patients (16–18); however, findings across studies are heterogeneous, and few have simultaneously assessed neural activity synchrony, activity intensity, and NVC efficiency. More importantly, whether structural damage, hypoperfusion, neural activity abnormalities, and NVC uncoupling exhibit region-specific associations, and how they collectively drive cognitive decline, remains to be systematically investigated through multimodal evidence.

On this basis, we hypothesized that in T2DM patients, key nodes of the Papez circuit and limbic system—including the hippocampus, anterior thalamic nuclei, and amygdala—exhibit not only volume atrophy and hypoperfusion, but also abnormal neural activity synchrony and reduced NVC efficiency, and that these multimodal alterations are closely correlated with glycemic control and cognitive function. To test this hypothesis, the present study employed multimodal MRI to simultaneously acquire high-resolution structural images, arterial spin labeling (ASL) perfusion images, and resting-state functional MRI (rs-fMRI) in the same cohort of T2DM patients. By systematically comparing the differences in these multimodal metrics between T2DM patients and healthy controls, combined with correlation analyses with glycemic parameters and cognitive assessments, we aimed to delineate multi-level pathological features of T2DM-related cognitive decline and to provide imaging targets for early identification and intervention.

## 2 Materials and methods

### 2.1 Participants

Participants included patients from the Department of Endocrinology and individuals from the Health Examination Center at Zhanjiang Central People's Hospital between November 2022 and January 2024. This study was approved by the Institutional Ethics Committee of Zhanjiang Central People's Hospital (approval No. KY20220124-07). The study was conducted in accordance with the principles of the Declaration of Helsinki. All participants provided written informed consent before enrollment.

Patients with T2DM were diagnosed by endocrinologists according to the diagnostic criteria of the American Diabetes Association (ADA) (19). Healthy controls (HC) were individuals who underwent health checkups and were confirmed to be free of systemic diseases. All participants were right-handed, native Chinese-speaking Han Chinese individuals. The exclusion criteria were as follows: age < 30 or >65 years; organic central nervous system diseases; personal or family history of psychiatric disorders; history of severe head trauma; history of severe hypoglycemia; macrovascular complications; history of alcohol dependence or substance abuse; significant hearing or visual impairment; pregnancy, lactation, or current use of oral contraceptives (in females); and contraindications to MRI. General demographic data, including sex, age, and education level, were collected through self-report. A total of 49 T2DM patients and 43 HCs were enrolled in this study.

### 2.2 Glycemic parameters assessment

Biochemical parameters measured in T2DM patients included glycated hemoglobin (HbA1c) and fasting blood glucose (FBG).

### 2.3 Cognitive function tests

All participants underwent a battery of neuropsychological assessments, including the montreal cognitive assessment (MoCA), the digit span test (DST, including both forward and backward subtests), and the clock drawing test (CDT).

### 2.4 MRI acquisition

MRI data were acquired using a 3T GE SIGNA Pioneer MRI scanner equipped with a 16-channel phased-array head coil. Conventional T1-weighted, T2-weighted, and T2-fluid-attenuated inversion recovery (T2-FLAIR) images were first obtained as routine clinical MRI data to exclude any visible severe brain lesions. Subsequently, high-resolution three-dimensional T1-weighted imaging (3D-T1WI), three-dimensional arterial spin labeling (3D-ASL), and resting-state functional MRI (rs-fMRI) using an echo-planar imaging sequence were performed sequentially. The detailed scanning parameters are provided below:

3D-T1WI scanning parameters: flip angle = 12.0°, bandwidth = 31.25 kHz, TR = 7.8 ms, TE = 3.1 ms, slices = 1024.0, thickness = 1.0 mm, pixel size = 1.0 × 1.0 × 1.0 mm^3^, FOV = 25.6 × 25.6 cm^2^, and NEX = 1.

Rs-fMRI scanning parameters: flip angle = 90.0°, bandwidth = 250.0 kHz, TR = 2,000 ms, TE = 30.0 ms, slices = 48.0, thickness = 3.0 mm, pixel size = 3.0 × 3.0 × 3.0 mm^3^, FOV = 256 × 256 mm^2^, and NEX = 1.

3D-ASL scanning parameters: TR = 4,852 ms, TE = 12.1 ms, NEX = 3, slice thickness = 3 mm, FOV = 256 mm × 256 mm, Recon Matrix = 235, arms = 10, Acquisition points = 576, slices = 46, PLD = 2.0 s.

### 2.5 Image processing

High-resolution T1-weighted images were analyzed using FreeSurfer 7.3 [Athinoula A. Martinos Center for Biomedical Imaging (under the Laboratory for Computational Neuroimaging), Boston, USA] to calculate the volumes of the bilateral hippocampus, amygdala, anterior thalamus, caudate nucleus, nucleus accumbens, putamen, and globus pallidus.

CBF maps were generated from the 3D-ASL images of each participant using GE's ADW4.5 post-processing software. The CBF maps were aligned to standard space using the SPM12 toolbox (implemented in MATLAB) and a standard ASL template.

rs-fMRI images were preprocessed using SPM12 (Wellcome Department of Imaging Neuroscience, University College London, London, UK) and DPABI (version 8.2; Institute of Psychology, Chinese Academy of Sciences, Beijing, China) toolboxes following established pipelines (20). The rs-fMRI preprocessing steps were as follows: the 3D T1 structural images and rs-fMRI functional images in DICOM format were first converted to NIFTI format, and the first 10 time points were discarded to allow for signal stabilization. Subsequently, slice timing and head-motion correction were performed; participants with translational head motion > 2 mm or rotational head motion >2° were excluded, and the Friston 24 head-motion parameters were calculated. Nuisance covariates, including linear drift, head-motion parameters, white matter signal, and cerebrospinal fluid signal, were regressed out, and the functional images were normalized to the MNI space using DARTEL. Finally, signals within the frequency band of 0.01–0.1 Hz were retained. After preprocessing, fractional amplitude of low-frequency fluctuations (fALFF), regional homogeneity (ReHo), and degree centrality (DC) maps were computed. fALFF was calculated prior to band-pass filtering using the 0.01–0.1 Hz frequency band, while DC maps were generated using a correlation coefficient threshold of *r* = 0.25 (the default value of the post-processing software). Z-score transformation was applied to improve the normality of the parameters. No spatial smoothing was performed to preserve the voxel-specific information required for subsequent NVC calculation.

For each participant, CBF values and functional MRI metrics (fALFF, DC, and ReHo) were extracted for the hippocampus, amygdala, anterior thalamus, caudate nucleus, nucleus accumbens, putamen, and globus pallidus using the Tian2020_Subcortex_Atlas (The University of Melbourne, Melbourne, Australia) template (21).

Within each of the aforementioned brain regions, voxel-wise CBF values were correlated with fALFF values, and the resulting Pearson correlation coefficient was defined as the the resting-state NVC value of that region. Note that this resting-state CBF–fALFF correlation reflects the efficiency of steady-state metabolic support for spontaneous neural activity, which is distinct from classical stimulus-evoked neurovascular coupling. Nevertheless, as a valid surrogate marker of neurovascular integrity, it has been widely adopted in clinical neuroimaging studies (5–7).

### 2.6 Statistical analysis

Statistical analyses were performed using SPSS (IBM SPSS, version 25; IBM (which acquired the original developer SPSS Inc. in 2009), Chicago, USA). For intergroup comparisons of continuous variables, independent-samples *t*-tests were used for data conforming to normal distribution; otherwise, the Mann–Whitney *U* test was applied. Categorical variables were compared using the chi-square test. The significance level was set at *P* < 0.05.

Analysis of covariance (ANCOVA) was performed to compare subcortical volumes, CBF, functional MRI metrics, and NVC values between groups. Sex, age, and education level were included as covariates in the comparisons of volume and CBF. For comparisons of functional MRI metrics and NVC, mean head-motion parameters were additionally included as covariates (mean head-motion parameters were only required when processing BOLD signals). To ensure reliability, the Benjamini–Hochberg (B-H) procedure was applied for false discovery rate (FDR) correction to control for multiple comparisons, with significance defined as a corrected *P*-value (i.e., Q value) < 0.05.

Within the T2DM group, partial correlation analyses were conducted to explore the associations between MoCA scores, glycemic metabolic measurements, and imaging metrics in regions showing significant intergroup differences. Statistical significance was set at *P* < 0.05. The covariates used were consistent with those in the intergroup comparisons.

## 3 Results

### 3.1 Demographics and cognitive assessments

No significant differences were observed between the two groups in terms of sex, age, or education level. The T2DM group exhibited significantly lower MoCA scores compared with the healthy control group (*P* < 0.05). No significant differences were found between the two groups in DST or CDT (Table 1).

**Table 1:** Demographic data, clinical biochemical indicators and neuropsychological result of all subjects.

Characteristic	T2DM (*n*= 49)	HC (*n* =43)	Statistics	*p*-value
Age (years)	52.78 ± 9.66	49.23 ± 8.00	*T* = 1.901	0.061
Sex (female/male)	22/27	25/18	*χ^2^* = 1.617	0.205
Education (years)	12.00 (9.00; 15.00)	12.00 (9.00; 16.00)	*z* = −0.889	0.374
HbA1c (%)	8.86 (7.13; 9.94)	5.42 (5.32; 5.53)	*z* = −7.887	< 0.001^*^
FBG (mmol/L)	8.15 (7.30; 11.50)	5.19 (4.91; 5.40)	*z* =-7.353	< 0.001^*^
MoCA	26 (22.50; 27.00)	27.00 (26.00; 28.00)	*z* = −3.590	< 0.001^*^
DST (forward)	8.00 (7.00; 9.00)	8.00 (8.00;9.00)	*z* = −1.071	0.284
DST (inverse)	4.00 (3.00; 4.00)	4.00 (3.00; 5.00)	*z* = −1.678	0.093
CDT	3.00 (3.00; 4.00)	4.00 (3.00; 4.00)	*z* = −1.406	0.160

FBG, fasting blood glucose; MoCA, Montreal Cognitive Assessment; DST, the digital span test; CDT, clock drawing test; ^*^p < 0.05.

### 3.2 Imaging metrics of the hippocampus and subcortical structures

Volume analysis: The right anterior thalamic volume was significantly smaller in the T2DM group than in the healthy control group (*P* < 0.05). No significant differences were observed in the volumes of the left anterior thalamus, bilateral hippocampus, amygdala, caudate nucleus, nucleus accumbens, putamen, or globus pallidus (Figure 1A).

**Figure 1:**
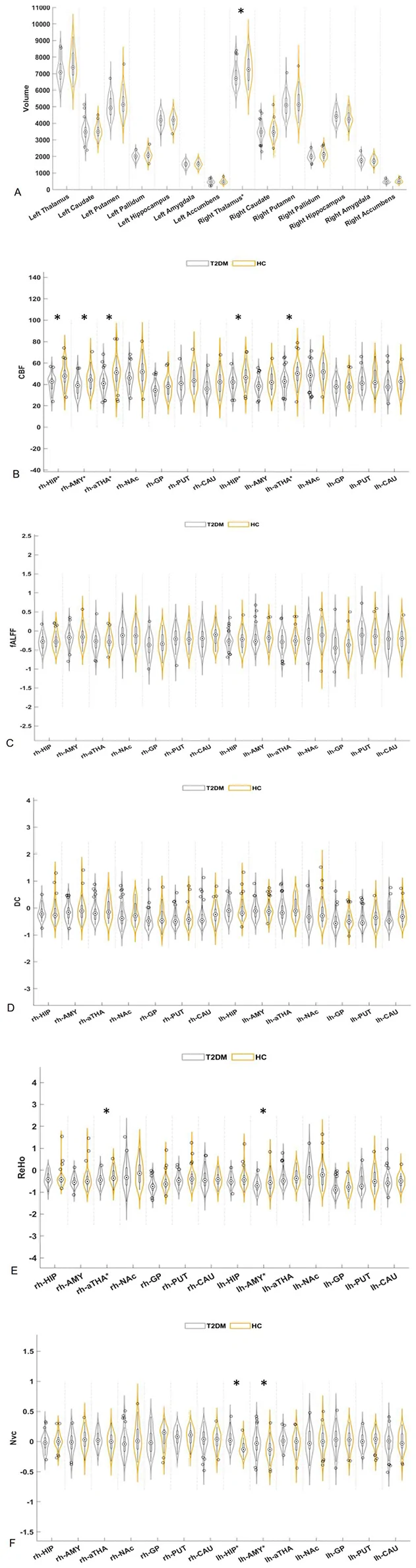
The right anterior thalamic volume was significantly smaller in the T2DM group than in the HC group **(A)**; CBF values in the bilateral hippocampus, anterior thalamus, and right amygdala were significantly lower in the T2DM group than in the HC group **(B)**; no significant differences were observed in fractional amplitude of low-frequency fluctuations (fALFF) **(C)** or degree centrality (DC) **(D)** between the two groups; ReHo values in the right anterior thalamus and left amygdala were lower in the T2DM group than in the HC group **(E)**; and NVC values in the left hippocampus and left amygdala were lower in the T2DM group than in the HC group **(F)**. ^*^P < 0.05.

Cerebral blood flow analysis: CBF values in the bilateral hippocampus, bilateral anterior thalamus, and right amygdala were significantly lower in the T2DM group than in the healthy control group (all *P* < 0.05). No significant differences were found in CBF values of the left amygdala, bilateral caudate nucleus, nucleus accumbens, putamen, or globus pallidus (Figure 1B).

Functional metrics analysis: No significant differences were observed between the two groups in fALFF or DC of the bilateral hippocampus, amygdala, anterior thalamus, caudate nucleus, nucleus accumbens, putamen, or globus pallidus (Figures 1C, D). ReHo values in the right anterior thalamus and left amygdala were significantly lower in the T2DM group than in the healthy control group (all *P* < 0.05). No significant differences were found in ReHo values of the left anterior thalamus, right amygdala, bilateral hippocampus, caudate nucleus, nucleus accumbens, putamen, or globus pallidus (Figure 1E).

NVC analysis: NVC values in the left hippocampus and left amygdala were significantly lower in the T2DM group than in the healthy control group (all *P* < 0.05). No significant differences were observed in NVC values of the right hippocampus, right amygdala, bilateral anterior thalamus, caudate nucleus, nucleus accumbens, putamen, or globus pallidus (Figure 1F). Detailed imaging metric values for the hippocampus and subcortical structures are presented in Table 2.

**Table 2:** Imaging indicators of bilateral hippocampus and subcortical structures in all subjects.

Imaging indicators	T2DM (*n* = 49)	HC (*n* = 43)	Statistics (*F*)	*p*-value	*q*-value
Volume of bilateral hippocampus and subcortical structures					
Left thalamus	7181.94 ± 703.11	7567.07 ± 818.81	5.178	0.025	0.118
Left caudate	3491.63 ± 552.11	3526.73 ± 388.29	0.520	0.473	0.827
Left putamen	4993.55 ± 588.31	5199.46 ± 723.27	2.884	0.093	0.260
Left pallidum	1995.58 ± 195.05	2091.40 ± 274.98	4.509	0.037	0.128
Left hippocampus	4174.40 ± 357.22	4159.38 ± 354.11	0.051	0.822	0.885
Left amygdala	1534.01 ± 177.05	1545.91 ± 181.80	0.064	0.801	0.935
Left accumbens	460.67 ± 109.11	480.60 ± 106.87	0.085	0.772	0.982
Right thalamus	6842.80 ± 646.91	7253.66 ± 724.93	9.746	0.002	0.034^*^
Right caudate	3482.05 ± 499.90	3501.97 ± 478.14	0.325	0.570	0.887
Right putamen	5105.72 ± 605.70	5232.27 ± 706.76	0.834	0.364	0.727
Right pallidum	2006.60 ± 223.75	2120.32 ± 251.06	6.422	0.013	0.091
Right hippocampus	4415.82 ± 358.85	4324.99 ± 404.67	1.523	0.220	0.514
Right amygdala	1744.08 ± 196.34	1745.74 ± 193.62	0.021	0.884	0.884
Right accumbens	477.07 ± 90.00	498.05 ± 87.31	0.242	0.624	0.874
CBF of bilateral hippocampus and subcortical structures					
rh-HIP	41.24 ± 7.65	48.42 ± 10.38	9.781	0.002	0.011^*^
rh-AMY	38.29 ± 7.71	44.05 ± 9.06	7.167	0.009	0.031^*^
rh-aTHA	41.00 ± 9.06	49.98 ± 12.30	12.270	0.001	0.010^*^
rh-NAc	45.88 ± 9.27	52.11 ± 11.31	4.472	0.037	0.087
rh-GP	34.72 ± 7.01	37.13 ± 8.07	1.322	0.253	0.322
rh-PUT	41.01 ± 8.62	45.45 ± 10.02	3.048	0.084	0.148
rh-CAU	36.96 ± 8.09	41.99 ± 9.75	3.066	0.083	0.167
lh-HIP	41.37 ± 8.12	47.78 ± 10.21	6.917	0.010	0.028^*^
lh-AMY	39.02 ± 7.07	42.20 ± 8.18	2.121	0.149	0.232
lh-aTHA	42.22 ± 9.26	50.34 ± 10.98	11.437	0.001	0.008^*^
lh-NAc	47.80 ± 9.72	51.61 ± 9.84	1.618	0.207	0.289
lh-GP	36.93 ± 7.94	37.60 ± 7.94	0.115	0.735	0.735
lh-PUT	41.58 ± 8.22	44.24 ± 9.77	0.915	0.342	0.368
lh-CAU	38.98 ± 8.61	42.33 ± 9.09	1.025	0.314	0.366
fALFF of bilateral hippocampus and subcortical structures					
rh-HIP	−0.30 ± 0.18	−0.27 ± 0.21	0.163	0.688	0.963
Rh-AMY	−0.19 ± 0.23	−0.15 ± 0.24	0.433	0.512	1.195
Rh-aTHA	−0.30 ± 0.23	−0.25 ± 0.21	0.935	0.336	1.569
rh-NAc	−0.11 ± 0.28	−0.11 ± 0.26	0.243	0.624	1.247
rh-GP	−0.38 ± 0.28	−0.33 ± 0.26	0.587	0.446	1.560
rh-PUT	−0.21 ± 0.25	−0.20 ± 0.24	0.008	0.928	0.928
rh-CAU	−0.21 ± 0.25	−0.13 ± 0.25	1.123	0.292	2.046
lh-HIP	−0.27 ± 0.21	−0.24 ± 0.23	0.065	0.800	0.933
lh-AMY	−0.20 ± 0.27	−0.15 ± 0.23	0.221	0.640	1.120
lh-aTHA	−0.29 ± 0.26	−0.23 ± 0.23	1.135	0.290	4.054
lh-NAc	−0.16 ± 0.29	−0.11 ± 0.30	0.033	0.857	0.923
lh-GP	−0.40 ± 0.28	−0.37 ± 0.27	0.174	0.678	1.055
lh-PUT	−0.14 ± 0.28	−0.14 ± 0.27	0.452	0.503	1.409
lh-CAU	−0.23 ± 0.28	−0.19 ± 0.28	0.131	0.719	0.915
DC of bilateral hippocampus and subcortical structures					
rh-HIP	−0.18 ± 0.25	−0.16 ± 0.39	0.249	0.619	0.788
rh-AMY	−0.15 ± 0.31	−0.08 ± 0.40	0.528	0.470	0.822
rh-aTHA	−0.16 ± 0.35	−0.07 ± 0.36	1.755	0.189	1.321
rh-NAc	−0.28 ± 0.41	−0.18 ± 0.38	0.524	0.471	0.733
rh-GP	−0.45 ± 0.31	−0.36 ± 0.37	1.080	0.302	0.845
rh-PUT	−0.43 ± 0.30	−0.36 ± 0.34	0.409	0.524	0.734
rh-CAU	−0.35 ± 0.39	−0.21 ± 0.35	2.270	0.136	1.897
lh-HIP	−0.08 ± 0.29	−0.08 ± 0.38	0.042	0.839	0.839
lh-AMY	−0.10 ± 0.31	−0.07 ± 0.33	0.152	0.697	0.814
lh-aTHA	−0.10 ± 0.38	−0.02 ± 0.36	1.255	0.266	1.240
lh-NAc	−0.22 ± 0.42	−0.14 ± 0.47	0.129	0.720	0.775
lh-GP	−0.50 ± 0.33	−0.43 ± 0.30	1.132	0.290	1.016
lh-PUT	−0.44 ± 0.32	−0.36 ± 0.32	0.690	0.408	0.817
lh-CAU	−0.34 ± 0.37	−0.26 ± 0.33	0.707	0.403	0.940
ReHo of bilateral hippocampus and subcortical structures					
rh-HIP	−0.39 ± 0.28	−0.38 ± 0.39	0.021	0.885	1.033
rh-AMY	−0.51 ± 0.25	−0.41 ± 0.46	1.960	0.165	0.289
rh-aTHA	−0.43 ± 0.29	−0.26 ± 0.32	8.260	0.005	0.036^*^
rh-NAc	−0.22 ± 0.58	−0.13 ± 0.52	0.342	0.560	0.784
rh-GP	−0.73 ± 0.30	−0.57 ± 0.38	3.522	0.064	0.224
rh-PUT	−0.46 ± 0.28	−0.35 ± 0.42	1.531	0.219	0.341
rh-CAU	−0.42 ± 0.42	−0.41 ± 0.29	0.034	0.854	1.086
lh-HIP	−0.51 ± 0.26	−0.39 ± 0.39	3.941	0.050	0.235
lh-AMY	−0.71 ± 0.27	−0.52 ± 0.38	10.000	0.002	0.030^*^
lh-aTHA	−0.39 ± 0.38	−0.31 ± 0.33	2.092	0.152	0.303
lh-NAc	−0.25 ± 0.57	−0.20 ± 0.55	0.019	0.891	0.960
lh-GP	−0.84 ± 0.34	−0.74 ± 0.31	2.174	0.144	0.336
lh-PUT	−0.60 ± 0.39	−0.43 ± 0.44	3.077	0.083	0.232
lh-CAU	−0.51 ± 0.38	−0.50 ± 0.26	0.000	0.985	0.985
NVC of bilateral hippocampus and subcortical structures					
rh-HIP	−0.02 ± 0.14	0.01 ± 0.12	1.587	0.211	0.369
rh-AMY	−0.02 ± 0.17	0.04 ± 0.16	2.218	0.140	0.327
rh-aTHA	0.03 ± 0.13	0.00 ± 0.14	2.457	0.121	0.422
rh-NAc	−0.02 ± 0.21	0.05 ± 0.23	1.614	0.207	0.415
rh-GP	0.00 ± 0.20	0.09 ± 0.17	6.368	0.013	0.063
rh-PUT	0.07 ± 0.13	0.09 ± 0.13	0.005	0.946	0.946
rh-CAU	0.03 ± 0.18	0.02 ± 0.14	0.667	0.416	0.648
lh-HIP	0.03 ± 0.13	−0.11 ± 0.11	34.006	0.000	< 0.000^*^
lh-AMY	−0.02 ± 0.19	−0.14 ± 0.16	8.005	0.006	0.041^*^
lh-aTHA	0.01 ± 0.12	−0.03 ± 0.16	2.374	0.127	0.356
lh-NAc	0.00 ± 0.20	0.01 ± 0.19	0.230	0.633	0.805
lh-GP	0.03 ± 0.20	0.03 ± 0.16	0.028	0.866	1.011
lh-PUT	0.02 ± 0.13	0.03 ± 0.14	0.302	0.584	0.818
lh-CAU	−0.02 ± 0.19	0.00 ± 0.16	0.005	0.943	1.015

HIP, Hippocampus; AMY, Amygdala; aTHA, Anterior thalamus; NAc, Nucleus accumbens; GP, Globus pallidus; PUT, Putamen; CAU, Caudate; ^*^q < 0.05.

### 3.3 Partial correlation analyses

Within the T2DM group, MoCA scores were negatively correlated with FBG (*r* = −0.405, *P* = 0.005) and positively correlated with right thalamic volume (*r* = 0.311, *P* = 0.035). Both HbA1c and FBG were negatively correlated with CBF in the left anterior thalamus (*r* = −0.344, *P* = 0.019; and *r* = −0.367, *P* = 0.012, respectively) (Figure 2).

**Figure 2:**
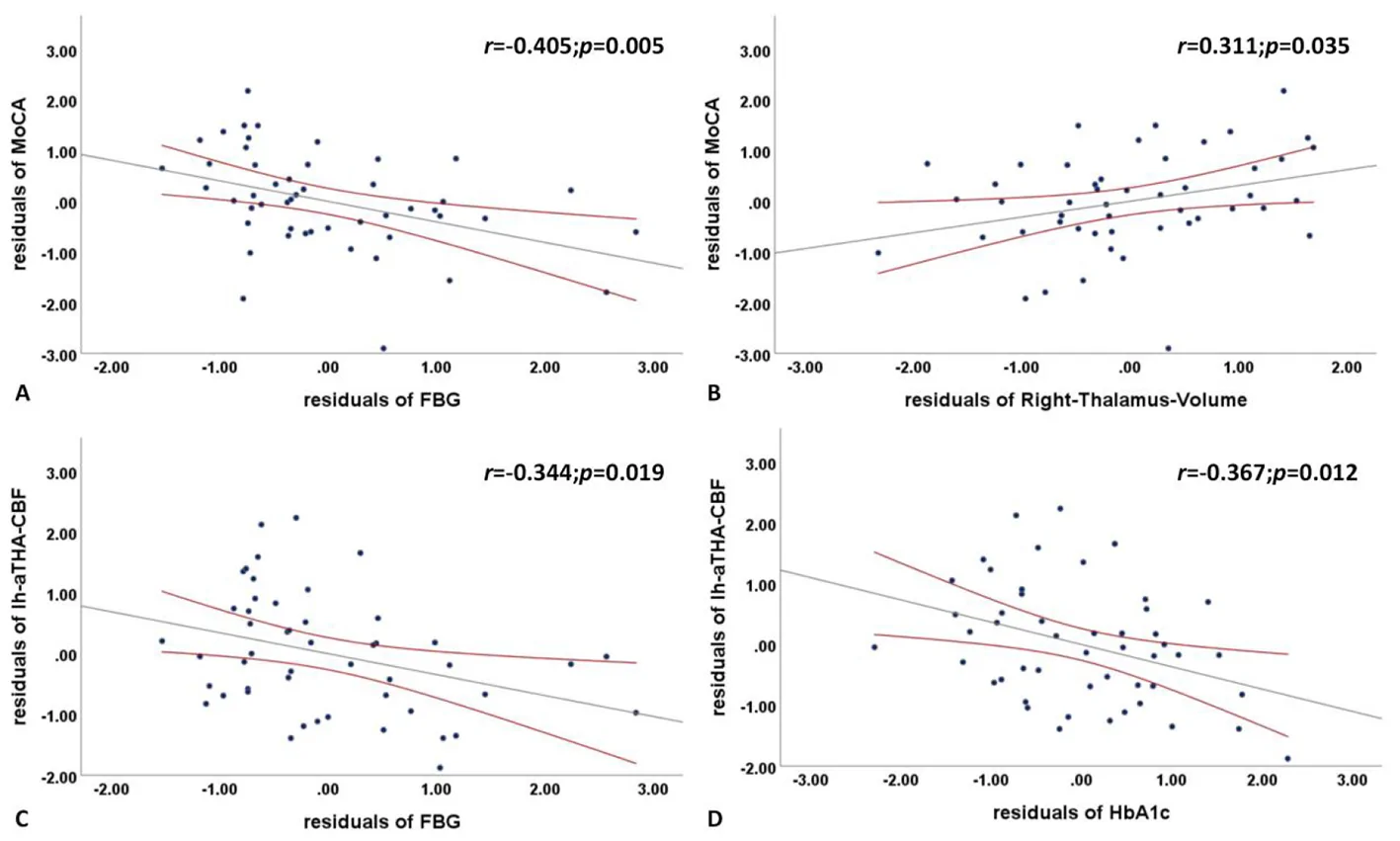
Correlations among cognitive function, clinical biochemical indices, and imaging metrics in the bilateral hippocampus and subcortical structures in T2DM patients. MoCA score was negatively correlated with FBG **(A)**. MoCA score was positively correlated with right thalamic volume **(B)**. FBG was negatively correlated with CBF in the left anterior thalamus **(C)**. HbA1c was negatively correlated with CBF in the left anterior thalamus **(D)**.

## 4 Discussion

The significantly lower MoCA scores in the T2DM group reflect a statistical between-group difference in cognitive function and do not imply that our sample meets the clinical diagnostic criteria for MCI. A definitive MCI diagnosis requires comprehensive neuropsychological assessments and age- and education-adjusted normative data, whereas the optimal MoCA cut-off in T2DM populations remains debated. We therefore define this finding as “T2DM-related cognitive decline,” in contrast to “T2DM with MCI.”

In the present study, CBF was significantly decreased in the bilateral hippocampus, bilateral anterior thalamus, and right amygdala in T2DM patients, whereas volume atrophy was observed only in the right anterior thalamus. This pattern of “widespread perfusion changes but restricted structural changes” suggests that CBF reduction may be a more sensitive hemodynamic biomarker than macroscopic volume atrophy in the course of T2DM-related brain injury. However, it must be emphasized that the cross-sectional design of this study precludes direct inference of the temporal relationship between CBF decline and volume atrophy. Alternative explanations for this pattern include the possibility that right anterior thalamic volume atrophy may be influenced by individual congenital anatomical variations (22), or that hypoperfusion and volume atrophy actually occur concurrently, with the former being more readily detected at the cross-sectional level due to the higher statistical sensitivity of CBF measurements compared with volumetric measurements (or larger effect sizes) (23, 24). Therefore, we speculate that CBF changes may precede the appearance of gross structural atrophy, whereas the manifestation of macroscopic volume atrophy might require a longer disease duration or more severe metabolic disturbances to develop—a speculation that awaits validation by longitudinal follow-up studies.

Although no significant differences were observed between the two groups in fALFF, which reflects the intensity of neural activity, or in DC, which reflects network centrality, T2DM patients exhibited significantly decreased ReHo in the right anterior thalamus and left amygdala. ReHo measures the synchrony of time series among neighboring voxels and represents the efficiency of local functional integration (25). The anterior thalamus is a key node of the Papez circuit and participates in the encoding and retrieval of episodic memory (11); the amygdala is responsible for emotional memory and emotional regulation (13). Decreased ReHo in these two regions implies impaired coordination of local neuronal activity, which may interfere with the efficiency of information transmission within the circuit. Notably, the regions with ReHo abnormalities partially overlapped with those showing CBF reductions, but the overlap was not complete, suggesting that although hypoperfusion and local synchrony abnormalities are closely related, there may be independent contributing factors or compensatory mechanisms.

One of the most central findings of this study is the significant reduction in NVC values in the left hippocampus and left amygdala in T2DM patients. NVC, defined as the voxel-wise correlation coefficient between regional CBF and fALFF, reflects the dynamic matching efficiency between neuronal activity and local blood supply (4, 26). In a healthy neurovascular unit, neuronal excitation rapidly triggers local vasodilation through the release of vasoactive substances by astrocytes, pericytes, and endothelial cells (27, 28). The decreased NVC observed in this study indicates that even at rest, cerebral blood flow in T2DM patients can no longer effectively follow spontaneous fluctuations in neural activity, resulting in neuro-vascular uncoupling. This uncoupling state may originate from chronic hyperglycemia-induced oxidative stress, endothelial nitric oxide synthase dysfunction, or pericyte loss. Notably, fALFF itself showed no intergroup differences, suggesting that the basic intensity of neural activity is maintained, but the blood flow regulatory mechanisms supporting its energy metabolism are already impaired. This provides a new perspective for understanding “subclinical” brain functional impairment in T2DM: conventional functional activity metrics may appear normal, whereas the decline in neurovascular coupling efficiency may emerge earlier.

The partial correlation analyses further support the above pathological chain. Within the T2DM group, poorer glycemic control was directly associated with hypoperfusion in the left anterior thalamus, identifying the anterior thalamus as a key target of hyperglycemic injury. Higher fasting blood glucose was linked to poorer cognitive performance, whereas larger right thalamic volume exerted a protective effect on cognition. The preservation of right thalamic volume may reflect relatively intact structural reserve, which buffers the negative impact of hyperglycemia on cognition. CBF in the left anterior thalamus was correlated with both glycemic indicators, whereas right thalamic volume was correlated only with MoCA scores rather than glycemic indicators, suggesting that volume atrophy may more reflect cumulative damage rather than immediate glycemic levels.

Notably, the lateralized injury pattern observed in this study—reduced NVC in the left hippocampus and amygdala, along with volumetric and CBF alterations in the right anterior thalamus—is not an isolated statistical phenomenon. Lateralized brain injury in T2DM has been previously reported: Milne et al. found that right hippocampal volume was negatively correlated with cognitive function (29), Cui et al. reported reduced left amygdala volume (30), and Hu et al. directly observed decreased neurovascular coupling in the left hippocampus and amygdala6, which was positively correlated with executive function—findings highly consistent with the present study. From the perspective of functional lateralization, the left hippocampus is primarily involved in verbal memory, while the right hippocampus is associated with non-verbal memory (31); emotion-related activation tends to be left-lateralized, and emotional memory is more strongly linked to left amygdala activation (32). Therefore, the left hippocampus-amygdala circuit may be more susceptible to T2DM-related metabolic stress due to its dominant role in verbal memory and emotional processing. The right anterior thalamus, as a key node of the Papez circuit, exhibits lateralized volumetric and CBF changes, suggesting a special vascular-structural vulnerability in this region; however, the underlying mechanisms remain to be elucidated. Currently, systematic evidence regarding lateralized injury of the anterior thalamus in T2DM remains limited, and the lateralized findings of this study warrant further validation through large-sample longitudinal follow-up investigations.

No significant intergroup differences were found in any metric—volume, CBF, ReHo, or NVC—in the basal ganglia structures, including the caudate nucleus, putamen, globus pallidus, and nucleus accumbens. This negative finding suggests that the impact of T2DM on brain structure and function is not diffuse global brain damage, but rather exhibits clear regional selectivity (33). Compared with the limbic system and Papez circuit nodes such as the hippocampus, amygdala, and anterior thalamus, the basal ganglia motor circuit appears to be more tolerant to chronic hyperglycemia. Possible explanations include: (1) the basal ganglia have relatively richer blood supply and better collateral circulation (34, 35); (2) the energy metabolic characteristics or vascular autoregulatory capacity of basal ganglia neurons may differ from those of the limbic system (36); and (3) the degree of cognitive decline in our participants was relatively mild (only MoCA declined, while DST and CDT were normal), and cognition- and emotion-related brain regions may be affected earlier than motor circuits (37). This finding also suggests that in the early stage of T2DM-related cognitive decline, brain damage may be concentrated in limbic-subcortical affective-memory circuits including the hippocampus, amygdala, and anterior thalamus, rather than involving all gray matter structures.

No significant differences in fALFF or DC were found between the T2DM group and the control group in this study, which is inconsistent with some previous reports. Potential reasons include: the relatively mild degree of cognitive decline in this patient cohort, in which overall neural activity intensity and global network centrality had not yet deviated significantly (38, 39); simultaneously, fALFF and DC reflect relatively global functional states and may have lower sensitivity to early changes than metrics reflecting local coordination or vascular coupling such as ReHo or NVC (40); furthermore, in the early stage of T2DM, it is possible that vascular regulatory function is impaired while neural activity can still be compensatorily maintained at normal levels, in which case NVC metrics best capture subclinical pathology (41, 42). These results suggest that in the mild stage of T2DM-related cognitive decline, selecting metrics sensitive to microvascular-neuronal coupling may have higher diagnostic value than global activity metrics.

Several limitations of this study should be acknowledged. First, the cross-sectional design precludes causal inference; for example, whether hypoperfusion is a cause or a consequence of impaired NVC requires longitudinal studies for verification. Second, the sample size was limited, and subgroup analyses based on disease duration or medication use were not performed, and these factors may have influenced the results. Third, due to the small size of subcortical nuclei, the limited resolution of ASL and fMRI may introduce partial volume effects and future studies should incorporate high-resolution perfusion imaging for validation. Fourth, voxel-wise correlations are constrained by spatial resolution and may be affected by noise, so the findings need to be corroborated by animal experiments. Fifth, our resting-state NVC reflects steady-state coupling rather than dynamic stimulus-evoked reactivity; although it is correlated with vascular reactivity measures, the two are not equivalent. Future studies should employ task-based fMRI combined with concurrent CBF measurements to directly compare static and dynamic neurovascular coupling. Sixth, the cross-sectional design and limited sample size of this study prevented subgroup stratification by disease duration, making it impossible to determine the temporal evolution sequence between CBF changes and volume atrophy. Future longitudinal follow-up studies or larger-sample stratified designs are needed to validate this chronological relationship. Seventh, we did not perform partial volume effect (PVE) correction for ASL-derived CBF. PVE may affect absolute CBF values; however, because both groups experienced systematic underestimation, the impact on between-group differences is likely limited. Future studies should apply PVE correction to further verify the robustness of our findings.

In conclusion, using multimodal MRI, this study revealed selective perfusion reduction, decreased local functional synchrony, and definitive neurovascular uncoupling in the hippocampus and key subcortical structures of T2DM patients, and demonstrated that these alterations are closely correlated with glycemic control and cognitive function. These findings provide multimodal imaging targets for early identification and intervention of T2DM-related cognitive decline.

## Data Availability

The raw data supporting the conclusions of this article will be made available by the authors, without undue reservation.

## References

[1] Kuate Defo A Bakula V Pisaturo A Labos C Wing SS Daskalopoulou SS. Diabetes, antidiabetic medications and risk of dementia: a systematic umbrella review and meta-analysis. Diabetes Obes Metab. (2024) 26:441–62. doi: 10.1111/dom.15331 37869901

[2] Crisóstomo J Duarte JV Canário N Moreno C Gomes L Castelo-Branco M. The longitudinal impact of type 2 diabetes on brain gyrification. Eur J Neurosci. (2023) 58:4384–92. doi: 10.1111/ejn.16177 37927099

[3] Dai P Yu Y Sun Q Yang Y Hu B Xie H . Abnormal changes of brain function and structure in patients with T2DM-related cognitive impairment: a neuroimaging meta-analysis and an independent validation. Nutr Diabetes. (2024) 14:91. doi: 10.1038/s41387-024-00348-5 39528442 PMC11554684

[4] Casey N Faraco G. Mechanisms of neurovascular regulation in health and disease: insights from experimental animal models. J Cereb Blood Flow Metab. (2026):271678x261448670. doi: 10.1177/0271678X261448670 42152599 PMC13186933

[5] Baller EB Valcarcel AM Adebimpe A Alexander-Bloch A Cui Z Gur RC . Developmental coupling of cerebral blood flow and fMRI fluctuations in youth. Cell Rep. (2022) 38:110576. doi: 10.1016/j.celrep.2022.110576 35354053 PMC9006592

[6] Xue H Xu X Yan Z Cheng J Zhang L Zhu W . Genome-wide association study of hippocampal blood-oxygen-level-dependent-cerebral blood flow correlation in Chinese Han population. iScience. (2023) 26:108005. doi: 10.1016/j.isci.2023.108005 37822511 PMC10562876

[7] Hu B Yan LF Sun Q Yu Y Zhang J Dai YJ . Disturbed neurovascular coupling in type 2 diabetes mellitus patients: evidence from a comprehensive fMRI analysis. NeuroImage Clinl. (2019) 22:101802. doi: 10.1016/j.nicl.2019.101802 30991623 PMC6447740

[8] Wang Y Sun L He G Gang X Zhao X Wang G . Cerebral perfusion alterations in type 2 diabetes mellitus - a systematic review. Front Neuroendocrinol. (2021) 62:100916. doi: 10.1016/j.yfrne.2021.100916 33957174

[9] Vetri F Xu H Paisansathan C Pelligrino DA. Impairment of neurovascular coupling in type 1 diabetes mellitus in rats is linked to PKC modulation of BK(Ca) and Kir channels. Am J Physiol Heart Circul Physiol. (2012) 302:H1274–84. doi: 10.1152/ajpheart.01067.2011 22268114 PMC3311480

[10] Duarte JV Guerra C Moreno C Gomes L Castelo-Branco M. Changes in hemodynamic response function components reveal specific changes in neurovascular coupling in type 2 diabetes. Front Physiol. (2022) 13:1101470. doi: 10.3389/fphys.2022.1101470 36703928 PMC9872943

[11] Hwang K Shine JM Cole MW Sorenson E. Thalamocortical contributions to cognitive task activity. eLife. (2022) 11:e81282. doi: 10.7554/eLife.81282 36537658 PMC9799971

[12] Rabiller G Ip Z Zarrabian S Zhang H Sato Y Yazdan-Shahmorad A . Type-2 diabetes alters hippocampal neural oscillations and disrupts synchrony between the hippocampus and cortex. Aging Dis. (2024) 15:2255–70. doi: 10.14336/AD.2023.1106 PMC11346393 38029397

[13] Fox AS Shackman AJ. An honest reckoning with the amygdala and mental illness. Am J Psychiatry. (2024) 181:1059–75. doi: 10.1176/appi.ajp.20240941 PMC11611071 39616453

[14] Ren X Zhang G Zhou B Gu W Jiang X Liao H . Revealing cerebral microvascular changes in diabetic rodents with ultrasound localization microscopy. Diabetes. (2025) 74:1469–79. doi: 10.2337/db25-0007 40633096

[15] Zhou J Zhang Z Zhou H Qian G. Diabetic cognitive dysfunction: from bench to clinic. Curr Med Chem. (2020) 27:3151–67. doi: 10.2174/1871530319666190206225635 30727866

[16] Moulton CD Costafreda SG Horton P Ismail K Fu CH. Meta-analyses of structural regional cerebral effects in type 1 and type 2 diabetes. Brain Imaging Behav. (2015) 9:651–62. doi: 10.1007/s11682-014-9348-2 25563229

[17] Liu J Yang X Li Y Xu H Ren J Zhou P. Cerebral blood flow alterations in type 2 diabetes mellitus: a systematic review and meta-analysis of arterial spin labeling studies. Front Aging Neurosci. (2022) 14:847218. doi: 10.3389/fnagi.2022.847218 35250549 PMC8888831

[18] Canário N Crisóstomo J Duarte JV Moreno C Quental H Gomes L . Irreversible atrophy in memory brain regions over 7 years is predicted by glycemic control in type 2 diabetes without mild cognitive impairment. Front Aging Neurosci. (2024) 16:1367563. doi: 10.3389/fnagi.2024.1367563 38590757 PMC10999637

[19] American Diabetes Association Professional Practice Committee. Classification and diagnosis of diabetes: standards of medical care in diabetes-2022. Diabetes Care. (2022) 45:S17–38. doi: 10.2337/dc22-S002 34964875

[20] Yan CG Wang XD Zuo XN Zang YF DPABI. Data processing & analysis for (resting-state) brain imaging. Neuroinformatics. (2016) 14:339–51. doi: 10.1007/s12021-016-9299-4 27075850

[21] Tian Y Margulies DS Breakspear M Zalesky A. Topographic organization of the human subcortex unveiled with functional connectivity gradients. Nat Neurosci. (2020) 23:1421–32. doi: 10.1038/s41593-020-00711-6 32989295

[22] Roshchupkin GV Gutman BA Vernooij MW Jahanshad N Martin NG Hofman A . Heritability of the shape of subcortical brain structures in the general population. Nat Commun. (2016) 7:13738. doi: 10.1038/ncomms13738 PMC5172387 27976715

[23] Li M Li Y Zhao K Tan X Chen Y Qin C . Changes in the structure, perfusion, and function of the hippocampus in type 2 diabetes mellitus. Front Neurosci. (2022) 16:1070911. doi: 10.3389/fnins.2022.1070911 36699515 PMC9868830

[24] Last D Alsop DC Abduljalil AM Marquis RP de Bazelaire C Hu K . Global and regional effects of type 2 diabetes on brain tissue volumes and cerebral vasoreactivity. Diabetes Care. (2007) 30:1193–9. doi: 10.2337/dc06-2052 17290035 PMC2031924

[25] Zang Y Jiang T Lu Y He Y Tian L. Regional homogeneity approach to fMRI data analysis. Neuroimage. (2004) 22:394–400. doi: 10.1016/j.neuroimage.2003.12.030 15110032

[26] Girouard H Iadecola C. Neurovascular coupling in the normal brain and in hypertension, stroke, and Alzheimer's disease. J Appl Physiol. (2006) 100:328–35. doi: 10.1152/japplphysiol.00966.2005 16357086

[27] Feng L Gao L. The role of neurovascular coupling dysfunction in cognitive decline of diabetes patients. Front Neurosci. (2024) 18:1375908. doi: 10.3389/fnins.2024.1375908 PMC10991808 38576869

[28] Koehler RC Gebremedhin D Harder DR. Role of astrocytes in cerebrovascular regulation. J Appl Physiol. (2006) 100:307–17. doi: 10.1152/japplphysiol.00938.2005 16357084 PMC1819408

[29] Milne NT Bucks RS Davis WA Davis TME Pierson R Starkstein SE . Hippocampal atrophy, asymmetry, and cognition in type 2 diabetes mellitus. Brain Behav. (2018) 8:e00741. doi: 10.1002/brb3.741 PMC5853633 29568674

[30] Cui D Liu X Liu M Cao W Xue Y Guo Y . Subcortical gray matter structural alterations in prediabetes and type 2 diabetes. Neuroreport. (2019) 30:441–5. doi: 10.1097/WNR.0000000000001224 30855559

[31] Burgess N Maguire EA O'Keefe J. The human hippocampus and spatial and episodic memory. Neuron. (2002) 35:625–41. doi: 10.1016/S0896-6273(02)00830-9 12194864

[32] Cahill L. Sex- and hemisphere-related influences on the neurobiology of emotionally influenced memory. Prog Neuropsychopharmacol Biol Psychiatry. (2003) 27:1235–41. doi: 10.1016/j.pnpbp.2003.09.019 14659478

[33] Moreno C d'Almeida OC Crisóstomo J Canário N Gomes L Castelo-Branco M. Habit-learning and decision-making circuits are susceptible to glycemic variability in type 2 diabetes: a longitudinal study. Front Neurosci. (2025) 19:1430185. doi: 10.3389/fnins.2025.1430185 40703625 PMC12283671

[34] Kuzucu P Çeltikçi P Demirtaş OK Canbolat Ç Çeltikçi E Demirci H . Arterial supply of the Basal Ganglia: a fiber dissection study. Operat Neurosurg. (2023) 24:e351–e9. doi: 10.1227/ons.0000000000000612 36719962

[35] Feekes JA Cassell MD. The vascular supply of the functional compartments of the human striatum. Brain. (2006) 129:2189–201. doi: 10.1093/brain/awl158 16815876

[36] Soares AF Nissen JD Garcia-Serrano AM Nussbaum SS Waagepetersen HS Duarte JMN. Glycogen metabolism is impaired in the brain of male type 2 diabetic Goto-Kakizaki rats. J Neurosci Res. (2019) 97:1004–17. doi: 10.1002/jnr.24437 31044444

[37] Hu B Yu Y Yu XW Ni MH Cui YY Cao XY . Sequence of episodic memory-related behavioral and brain-imaging abnormalities in type 2 diabetes. Nutr Diabetes. (2025) 15:1. doi: 10.1038/s41387-025-00359-w 39893169 PMC11787324

[38] Wang Q Hou C Jiang X Li H. Alterations of spontaneous brain activity in type 2 diabetes mellitus without mild cognitive impairment: a resting-state functional magnetic resonance study. Front Hum Neurosci. (2023) 17:1305571. doi: 10.3389/fnhum.2023.1305571 38273877 PMC10808360

[39] Liang Y Liu WV Li M Feng Q Wei S Yang W . Altered static and dynamic intrinsic brain activity patterns in type 2 diabetic patients. Sci Rep. (2025) 16:1142. doi: 10.1038/s41598-025-30847-z PMC12789552 41361357

[40] Zuo XN Di Martino A Kelly C Shehzad ZE Gee DG Klein DF . The oscillating brain: complex and reliable. Neuroimage. (2010) 49:1432–45. doi: 10.1016/j.neuroimage.2009.09.037 PMC2856476 19782143

[41] Yu Y Yan LF Sun Q Hu B Zhang J Yang Y . Neurovascular decoupling in type 2 diabetes mellitus without mild cognitive impairment: potential biomarker for early cognitive impairment. Neuroimage. (2019) 200:644–58. doi: 10.1016/j.neuroimage.2019.06.058 31252056

[42] Canário NS Crisóstomo J Moreno C Duarte JV Duarte IC Ribeiro MJ . Functional reorganization of memory processing in the hippocampus is associated with neuroprotector GLP-1 levels in type 2 diabetes. Heliyon. (2024) 10:e27412. doi: 10.1016/j.heliyon.2024.e27412 PMC10950584 38509913

